# Muscle-Bone Interactions in Chinese Men and Women Aged 18–35 Years

**DOI:** 10.1155/2020/8126465

**Published:** 2020-05-09

**Authors:** Meihua Su, Zhaojing Chen, Breanne Baker, Samuel Buchanan, Debra Bemben, Michael Bemben

**Affiliations:** ^1^College of Physical Education, Jimei University, Xiamen, Fujian, China; ^2^Neuromuscular Laboratory, Department of Health and Exercise Science, University of Oklahoma, Norman, OK, USA; ^3^Department of Kinesiology, California State University San Bernardino, San Bernardino, CA, USA; ^4^Bone Density Research Laboratory, Department of Health and Exercise Science, University of Oklahoma, Norman, OK, USA; ^5^Thompson Laboratory for Regenerative Orthopedics, Department of Orthopedic Surgery, University of Missouri, Columbia, MO, USA

## Abstract

To characterize bone mineral density (BMD), bone strength, muscle and fat mass, and muscle strength and power in Chinese women (*n* = 25) and men (*n* = 28) classified as in the bone accrual phase (18–25 years) or in the peak bone mass phase (26–35 years). Calcium intakes, physical activity levels, and serum vitamin D were measured. Dual-energy X-ray absorptiometry (DXA) assessed body composition, lumbar spine, and hip areal BMD (aBMD) variables and peripheral quantitative computed tomography (pQCT) assessed cortical and trabecular volumetric BMD (vBMD) and bone strength. Muscle strength and power were assessed by grip strength, leg press, and vertical jump tests. Calcium, serum vitamin D, and physical activity levels were similar across age and sex groups. Significant sex differences (*p* < 0.05) were found for most body composition variables, hip aBMD, tibia variables, and muscle strength and power. Adjusting for height and weight eliminated most of the significant sex differences. Women showed stronger positive correlations between body composition and bone variables (*r* = 0.44 to 0.78) than men. Also, correlations between muscle strength/power were stronger in women vs. men (*r* = 0.43 to 0.82). Bone traits were better related to body composition and muscle function in Chinese women compared to Chinese men aged 18 to 35 years, and peak bone mass seems to be achieved by 25 years of age in both Chinese men and women since there were no differences between the two age groups.

## 1. Introduction

Peak bone mass (usually achieved by 25 years of age) is essential to bone health and is associated with a potential risk of osteoporosis. Achieving peak bone mass is also a function of genetics and environmental factors including physical activity levels, muscle and fat mass (or bone loading patterns), and nutritional status (calcium intakes and vitamin D status) and may be related to sex and ethnicity [1–5]. Some racial/ethnicity differences in bone mineral density (BMD) for women are well documented. Asian women have a lower areal bone mineral density (aBMD), assessed by 2-dimensional dual-energy X-ray absorptiometry (DXA) scans, but higher volumetric bone mineral density (vBMD), obtained from 3-dimensional peripheral quantitative computed tomography (pQCT) scans that also differentiate cortical and trabecular bone, compared to Caucasian women [3, 6, 7]. In addition, Asian women have lower rates of hip and forearm fractures but similar risk for vertebral fractures as Caucasian women [8]. These incongruous findings are difficult to understand since it has been reported that both pre- and postmenopausal Chinese American women generally have a smaller bone size (DXA), yet thicker cortical and trabecular bone compartments (pQCT), and architecturally stronger bone, at least at the radius and tibia, and therefore display lower fracture rates at peripheral sites compared to Caucasians. However, one limitation to these findings is that these Chinese American women could have been born in the United States, moved from China, and lived in the United States for various lengths of time, or born in other countries before moving to the United States and not based on Chinese women, born and living in China for all their lives with the exception of the 5 or less years that they have lived in the United States as this study has established. Additionally, it is unknown if these findings also apply to Chinese men since they have been studied to a much lesser extent. Interestingly, premenopausal Chinese women also have been reported to have lower physical activity levels, lower dietary calcium intakes, and lower serum vitamin D concentrations compared to their Caucasian counterparts [5, 7].

There is controversial evidence regarding the contributions of DXA-derived bone-free lean body mass (BFLBM) or fat mass (FM) to bone health, specifically, which of these body composition variables exert greater influence on aBMD [9]. In the case of FM, the extra weight is hypothesized to load the skeleton through gravitational forces, thus increasing aBMD, whereas BFLBM contributes to both gravitational forces and forces from muscle contraction, thereby placing greater mechanical loads on the skeletal system yielding greater bone adaptations. Several studies have reported a positive association between BFLBM and bone mass [10, 11], but a number of other studies have demonstrated that both BFLBM and FM contribute equally to bone mass, especially in women [2, 12, 13]; however, most of those studies were carried out in Caucasian populations. Fewer studies have examined the factors associated with the bone health of Asian men or have explored sex differences in bone variables in Asians. Cheng et al. compared age and sex effects on aBMD and body composition in Chinese men and women and found that lean mass was the strongest predictor of aBMD at all ages for both Chinese men and women [14]. However, in general, little is known about the interactions between muscle mass and function (i.e., strength and power) and bone health in Chinese men and women and the variables that may influence these factors, such as vitamin D status, since vitamin D plays important roles in both calcium homeostasis and muscle function [15]. Therefore, the purpose of this investigation was to characterize bone mineral density, bone strength, muscle and fat mass, and muscle strength and power in 18- to 35-year-old Chinese women and men, either as they accrue bone mass (18–25 years) or have already obtained peak bone mass (26–35 years). A secondary purpose was to examine the relationships between DXA (aBMD) and pQCT (vBMD) variables and measures of body composition, muscle strength and power, and bone strength.

## 2. Materials and Methods

### 2.1. Study Participants

The current study included 25 female and 28 male Chinese adults aged 18 to 35 years who were divided into two groups: young (18–25 years) and still accruing bone mass and older (26–35 years), already achieved peak bone mass. All of the participants were international students or visiting scholars at the University of Oklahoma who came from China and had been in the USA for less than five years. All participants were normotensive and in good health according to resting blood pressures and the health status questionnaire, and none of the participants were taking any medications known to affect bone or soft tissue metabolism. All procedures performed were in accordance with the Ethical Standards of the University of Oklahoma and with the 1964 Helsinki Declaration and its later amendments or comparable ethical standards. Each subject read and signed a written informed consent approved by the Institutional Review Board (IRB#6202) at the University of Oklahoma, Norman, OK. Once enrolled in the study, subjects completed several questionnaires, including the Baecke Physical Activity Questionnaire [16], Bone-Specific Physical Activity Questionnaire (BPAQ) for the physical activities status in the past 12 months, and a validated calcium intake questionnaire [17] to estimate daily calcium intake from diet and supplements.

### 2.2. DXA Bone Status and Body Composition

All participants had their height, weight, and blood pressure measured before bone mineral density assessments. Height was measured with a stadiometer (Stadi-O-Meter, patent 290237, Novel Products, Rockton, IL), weight was measured with a digital electronic scale (BWB-800, Tanita Corporation of America, Arlington Heights, IL), and resting seated blood pressure was obtained after a 10-minute rest utilizing an OMRON blood pressure monitor (OMRON Healthcare Inc., Lake Forest, IL) placed on the left arm. A urine sample was also obtained from all subjects to measure specific gravity for hydration status, and a pregnancy test was performed for all women to ensure they were not pregnant prior to any scans. Dual-energy X-ray absorptiometry (DXA; GE Lunar-Prodigy, EnCore version 16) was used to measure the aBMD of the total body, anterior-posterior (AP) lumbar spine, dual proximal femur (femoral neck, trochanter, and total hip), and total body and regional measures of percent fat, FM, and BFLBM. Scan modes were determined by the subject's AP thickness as measured at the umbilicus by the software, and all scanning procedures were standardized for all subjects following the guidelines of the DXA manufacturer. Quality assurance procedures were performed daily, and the acquisition and analyses of all bone scans were performed by the same DXA technician in the Bone Density Research Laboratory. Coefficients of variation (CV) for precision and accuracy for the spine phantom are 0.6% and 0.8%, respectively. The in vivo precision and accuracy of the DXA root mean square (RMS) %CV for areal BMD is 0.7% for the total body BMD, 1.4% for the lumbar spine BMD, and 0.6% for total left and right hip, 0.6% for right trochanter, 0.7% for left trochanter, 0.9% for right femoral neck, and 1.01% for left femoral neck BMD. The International Society for Clinical Densitometry recommends that *Z*-scores ≤− 2.0 are considered below the expected range for age [18]. The *in vivo* precision of DXA RMS %CV for body composition variables is 2.0% for percent body fat and fat mass and 1.9% for BFLBM.

### 2.3. pQCT Bone Status

Volumetric bone mineral density (vBMD), bone strength, and geometry of the nondominant tibia were evaluated using pQCT. Subjects had three pQCT scans performed for the nondominant tibia (4%, 38%, and 66% sites of the tibia length) using a pQCT scanner XCT 3000 with software version 6.00 (Stratec Medizintechnik GmbH, Pforzheim, Germany) by a single technician. Bone strength variables included bone strength index (BSI) at the 4% site, and strength-strain index (SSI), a measure of torsional stiffness, at the 38% and 66% sites. The muscle cross-sectional area (MCSA) was assessed at the tibia 66% site. Analysis modes and thresholds were set to separate cortical bone from trabecular bone. The precision (RMS CV%) for pQCT bone variables ranges from 0.31 to 1.21% for all sites. The MCSA RMS CV% is 1.73%.

### 2.4. Muscle Strength and Power

Muscle power was assessed by a jump test on a jump mat (Just Jump, Probotic, AL) with a Tendo FITRODYNE power and speed analyzer (Tendo Sports Machines, Trencin, Slovak Republic). Subjects performed a countermovement vertical jump by crouching, then jumping with nonrestricted arm motion, and then landing on the jump mat. A minimum of 1-minute rest was allowed between jumps and trained spotters stood on either side of the subject to help with balance. A total of three successful jumps were performed for each subject, and the average was used in the data analyses. Average muscle power was estimated from the average force and velocity reported by the Tendo machine. The ICC values for jump power, time in air, jump height, and velocity range between 0.80 and 0.98.

Upper body muscle strength was assessed using a handgrip dynamometer (Takei Scientific Instruments, Yashiroda, Japan). Subjects sat in a chair with their back supported, the right elbow flexed at 90°, forearm in a neutral position, and wrist between 0° and 30° dorsiflexion and 0°–15° ulnar deviation. Grip width was adjusted to be comfortable for each subject, and subjects were encouraged to squeeze as hard as possible for about 3–5 seconds. The same measurement was repeated for the other hand (3 trials on each side, each separated by 1 min of rest). The highest maximal handgrip strength for right and left hands was used in the data analyses. The ICC for handgrip dynamometry is 0.874.

Two-leg muscle strength was assessed by a standard 1-repetition maximum test (1RM) on a semireclined Cybex two-leg press machine. This muscular strength testing procedure has been found to be reliable in our laboratory, with ICCs > 0.91 [19].

### 2.5. Blood Sampling and Biochemical Assay

Venipuncture blood draws (about 7 ml) for each subject were obtained by a registered nurse or phlebotomist at Oklahoma University Goddard Health Center in the morning after overnight fasting to measure serum vitamin D concentrations. The serum was stored at −84°C, and serum levels of 25-hydroxy vitamin D were measured in duplicate using an enzyme-linked immunosorbent assay (ELISA—Immunodiagnostic Systems Inc., USA). Intra-assay CVs were 0.67–7.87% and the interassay CVs were 0.04–1.25%.

### 2.6. Data Analyses

All data are reported as mean ± standard deviation (SD). SPSS version 24.0 (SPSS, Inc., Chicago, IL) was used to execute all statistical analyses. A two-way analysis of variance (ANOVA) was used to compare areal and volumetric BMD, regional and total body measures of FM, BFLBM, and MCSA, lower extremity jump power, and muscle strength variables between the two sexes (male and female) and the two age groups (bone accrual, younger (18–25 years) and peak bone mass, older (26–35 years)) before, and after, adjusting for height and weight differences with analysis of covariance (ANCOVA). Pearson correlation coefficients were used to determine relationships between measures of BMD and bone strength and age, height, body composition, and muscle performance measures. Statistical significance was set a priori at *p* ≤ 0.05.

## 3. Results

As expected, age was significantly different (*p* ≤ 0.01) between the younger (bone accrual) and the older (peak bone mass) groups as designed (Table 1). Males had significantly (*p* ≤ 0.01) greater height, weight, and resting systolic blood pressure (although still considered normotensive) than females which provided the basis for the follow-up ANCOVAs that made adjustments for height and body weight differences. Body mass index (BMI) was in the normal range for both age groups and sexes (18.5–24.9 kg/m^2^—underweight to normal or healthy weight), there were no age or sex differences in physical activity levels based on BPAQ scores and total METs expended and both were considered normal, calcium intakes were similar for all groups but below the expected 1000 mg/day except for the younger males (1012.9 mg/day), and average vitamin D levels were in the normal range (20–50 ng/ml) except for the younger females (18.9 ± 6.6 ng/ml) which would be considered inadequate for normal bone health. When examining individual subject vitamin D levels, it should be noted that there were 10 females in the younger age group (11.70–17.67 ng/ml), 4 females in the older age group (14.84–18.66 ng/ml), 5 males in the younger age group (12.33–19.40 ng/ml), and 3 males in the older age group (13.72–16.57 ng/ml) that were below the normal range (20–50 ng/ml). No significant sex × age interactions were observed.

### 3.1. Body Composition

Table 2 presents the total and regional measures of percent fat, FM, and BFLBM obtained by DXA. Interestingly, there were no age main effects for any of the variables of interest, but there were several sex main effects. Females had significantly (*p* ≤ 0.01) greater body percent fat (total body, arm, leg, and trunk) compared to males. Females also had significantly greater amounts of total body FM (*p* ≤ 0.05), arm FM (*p* ≤ 0.05), and leg FM (*p* ≤ 0.01) compared to males, whereas males had significantly (*p* ≤ 0.01) greater BFLBM (total body, arm, leg, and trunk) than females.

### 3.2. Areal Bone Mineral Density Measures (DXA)

Based on the initial two-way ANOVA, there were no significant (*p* ≤ 0.05) age or sex main effects for the BMD of the lumbar spine (L1–L4) but males had significantly (*p* ≤ 0.01) greater BMD at the femoral neck, trochanter, and total hip sites for both legs (Table 3). However, after adjusting for height and weight differences between males and females (two-way ANCOVA), females (both age groups combined) had a significantly (*p* ≤ 0.05) greater BMD at the L1–L4 lumbar region (before ANCOVA, males: 1.219 ± 0.084; females: 1.182 ± 0.146 g/cm^2^(*p* ≥ 0.05) vs. after ANCOVA, males: 1.148 ± 0.132; females: 1.257 ± 0.135 g/cm^2^(*p* ≤ 0.05)).

### 3.3. Volumetric Bone Mineral Density Measures (pQCT)

Measures of vBMD and bone strength at the standard 4%, 38%, and 66% of the tibia are presented in Table 4. Based on the initial two-way ANOVA, there was a significant (*p* ≤ 0.05) age main effect for cortical vBMD at the 66% tibial site, with the older group having greater values than the young group. There were also several significant (*p* ≤ 0.01) sex main effects. At the 4% tibial site, each variable of interest (total vBMD, trabecular vBMD, total BSI, and trabecular BSI) was significantly (*p* ≤ 0.01) greater in males compared to females. At the 38% tibial site, only the cortical SSI value was significantly (*p* ≤ 0.05) greater in males compared to females. No significant sex × age interactions were observed. After adjusting for height and weight differences between males and females (two-way ANCOVA), there were no significant age or sex main effects and no significant interactions for any of the vBMD or SSI variables.

### 3.4. Muscle Strength

Measures of muscle strength, power, and size (Table 5) were as expected, with males being significantly (*p* ≤ 0.01) stronger (1RM leg press and handgrip), having significantly (*p* ≤ 0.01) greater lower body muscle power (jump time, jump height, and jump power), and having significantly (*p* ≤ 0.01) larger MCSA at the tibia 66% site than females. Interestingly, older subjects (26–35 years) had a significantly (*p* ≤ 0.01) greater right handgrip strength and significantly (*p* ≤ 0.01) larger MCSA than the younger subjects (18–25 years). There was a significant sex × age interaction (*p*=0.04) for 1RM leg press with older men being stronger than younger men whereas there was no age difference for women. This finding remained after adjusting for height and weight (two-way ANCOVA).

### 3.5. Relationships

When evaluating the relationships between measures of size, mass, composition, muscle strength, muscle power, and bone strength and measures of bone mineral density in males (Table 6), overall, there were fewer and weaker significant correlation coefficients, than observed in females (Table 7). Of the 100 correlation coefficients calculated for the males, there were only 10 that were significant (6 were significant at *p* ≤ 0.01, ranging from 0.46 to 0.59, and 4 were significant at the *p* ≤ 0.05 levels, ranging from 0.38 to 0.45). Weight and max jump power had moderate, significant correlations with hip aBMD (*r* = 0.46–0.59, *p* ≤ 0.01) and 4% total BSI (*r* = 0.38–0.41, *p* ≤ 0.05). BFLBM showed a significant (*p* ≤ 0.05) low positive correlation only with femoral neck aBMD.

For females, 64 of the 100 calculated correlation coefficients were significant (48 at the *p* ≤ 0.01 level) and much stronger (most between 0.51 and 0.82) than those in the males. Most were positive and above 0.50, and most measures of size, mass, composition, muscle strength, and muscle power were related to hip BMD values (femoral neck, trochanter, and total hip) and tibia bone strength (BSI and SSI).

Body weight had moderate, significant correlations (*r* = 0.62–0.73; *p* ≤ 0.01) with L1–L4 and hip aBMD variables and moderate to strong correlations (*r* = 0.66–0.80, *p* ≤ 0.01) with vBMD and BSI at the 4% tibia site. Similarly, BMI, total percent fat, fat mass, and total BFLBM also had moderate, significant correlations (*r* = 0.44–0.69; *p* ≤ 0.05) with L1–L4 and hip aBMD and moderate to strong positive correlations (*r* = 0.45–0.78, *p* ≤ 0.05) with vBMD and BSI at the 4% site. These body composition variables generally were not significantly correlated with 38% and 66% cortical vBMD; however, BMI, total percent fat, and fat mass were positively correlated (*r* = 0.45–0.47; *p* ≤ 0.05) with 38% cortical SSI. Additionally, 1RM leg press and lower limb maximal power had moderate to strong significant correlations (*r* = 0.43–0.82; *p* ≤ 0.01) with measures of all aBMD and 4% tibia site variables and 38% cortical SSI.

To summarize the findings from the two-way ANOVAs, most significant main effects for age and sex disappeared when the data were adjusted for height and body weight (ANCOVA). There were also no significant interactions for any of the variables of interest, and there were no differences between groups for physical activity levels, calcium intakes, and vitamin D levels although many subjects were below normal calcium intakes and were below normal serum vitamin levels. Findings from the Pearson correlation coefficient analyses indicated that there were more significant relationships between measures of body composition, muscle performance, and bone status that were stronger for females compared to males.

## 4. Discussion

The aim of this cross-sectional study was to determine measures of bone health (aBMD, vBMD, and SSI) as they related to environmental factors (calcium intakes, vitamin D levels, physical activity levels, muscle and fat mass, and muscular strength and power), sex, and bone accrual status in an understudied ethnic group of native-born Chinese men and women aged 18–35 years. We had three unique findings: first, fat and muscle variables showed stronger correlations with BMD in women compared to men; second, most significant group differences in our outcome variables of bone, body composition, and muscle strength and power were independent of calcium intake, vitamin D levels, and physical activity levels and were based on sex rather than accrual phase or age, but became nonsignificant after accounting for height and body weight differences between the sexes; and third, the lack of age group difference in bone variables suggests that peak bone mass is achieved by 25 years of age in Chinese men and women. According to Heaney et al. [4], proximal femur sites peak before age 20 and the total body skeleton about 6–10 years later. This was the rationale for our age groups, between 18 and 25 years, the participants would likely still be accruing bone, especially for the total body, while 26 and older most would have completed the bone gains. In a large cohort of Chinese participants, Cheng et al. reported peak bone mass did not vary by site for men (all achieved between 20 and 29 years), but it occurred later for the spine and total body sites (30–39 years) than the femur site (20–29 years) in Chinese women [14]. It should be noted that Cheng et al. separated the age groups by decade, so it is possible that peak bone mass could have occurred at the midpoint of the decade (e.g., 25 or 35 years) [14].

Some racial differences in bone mineral density for women are well documented, with Asian women generally having lower aBMD but higher vBMD compared to Caucasian women [3, 5, 6, 20] often resulting in greater mechanical advantages and fewer osteoporotic fractures, at least in Chinese-American Women [6, 20]. Fewer studies have examined the bone health of Chinese men or have explored sex differences in bone variables in Chinese-born subjects.

It is also reasonably well established that achieving peak bone mass is a function of both genetic and environmental factors that include physical activity levels, muscle and fat mass, calcium intakes, and vitamin D levels and may be related to sex and ethnicity [1–4]. Among the environmental factors often examined in relation to bone health is body weight, which has been shown to be a strong and positive predictor of increased BMD based on studies with overweight individuals [21]. What is less clear is the impact that FM and BFLBM have on bone health [22]. Most agree that a strong relationship between BFLBM and bone structure exists, but data concerning the relationship between FM and BMD are not as well established. Studies have found contradictory results with FM being either positively or negatively associated with BMD and, to confuse the issue more, may be related to body location (spine, hip, and tibia).

Cheng et al. reported that lean mass was the strongest predictor of aBMD at all ages for both Chinese men and women [14]. Our findings do not support this finding in 18- to 35-year-old Chinese men and women. Only body weight had a moderate positive significant relationship with hip aBMD for the men in our study. In contrast, weight, BMI, FM, and BFLBM showed moderate positive significant correlations with spine and hip aBMD, and 4% tibia variables for women. The strong relationship with FM and bone health is similar to the study of Keska et al. who reported that FM was significant for BMD in young Polish women [23]. Also, Reddy et al. reported that BFLBM was an important determinant of BMD in men, but both BFLBM and FM were important in women aged 20–35 years [24].

It is generally agreed that environmental factors that are reflected in measures of body composition, physical activity levels, and diet play an important role in the attainment of peak BMD and bone health. To date, the interactions between muscle mass and function (e.g., strength and power) and bone health in Chinese young adults, particularly women, are understudied. Recently, both muscle strength and lean mass were reported to be positively correlated with spine, hip, and total body aBMD in Chinese men aged 20–47 years [25]. In contrast, muscle strength assessed by grip strength but not by muscle mass was related to bone status in an elderly cohort of Chinese men and women with higher grip strength being associated with a lower risk of osteoporosis [26]. Similar to our findings, Qi et al. found that muscle strength (grip strength) was positively associated with hip and spine aBMD in women (40–90 years) but not in their male counterparts [27]. However, they did find that muscle mass (relative appendicular skeletal muscle mass) was positively associated with aBMD in both sexes. These findings suggest that muscle strength may be important for bone status in Chinese women of all ages, whereas it may play a role in bone health in young but not in older Chinese men.

Regarding environmental factors mentioned above, like physical activity levels, calcium intakes, and vitamin D levels, young Chinese women have usually been found to be lower in these parameters compared to their white counterparts [5]. Our data suggest that there were no significant differences between males or females or between younger subjects in the bone accrual phase and older subjects who had already reached peak bone mass for calcium intakes, vitamin D levels, BPAQ totals, or total METs expended. Regarding physical activity levels, all BPAQ scores ranged between 22.0 and 26.4, with no differences across the 4 groups, and total MET expenditures from the Baecke Physical Activity Questionnaires were statistically the same for all 4 groups but were slightly higher for males (∼2850 METs) compared to females (∼2160 METs). These assessments were done to explore potential differences in physical activity patterns between age and sex that could influence bone health parameters; however, physical activity levels were similar across all groups and did not influence bone measures. Also, data from our study indicate that calcium intakes were similar for all groups but below the expected 1000 mg/day except for the younger males (1012.9 mg/day) and average vitamin D levels were in the normal range (20–50 ng/ml) except for the younger females (18.9 ± 6.6 ng/ml) which would be considered inadequate for normal bone health. Additionally, it should be noted that even though 40% of the younger and older females and 33% of the younger and 23% of the older males were below the normal range (20–50 ng/ml) for vitamin D levels, after adjusting for height and weight, there were no differences between age or sex for most of the bone parameters indicating no, or little, influence of low vitamin D levels. Our findings are similar to those reported by Henderson et al. in 18-year-old Caucasian women and their mothers which showed that weight and BFLBM, but not FM, correlated most consistently with BMD [28]. They also reported that physical activity levels as determined by questionnaires correlated weakly but significantly with BMD at the spine, hip, and trochanter sites but when weight was accounted for, it did not make a significant independent contribution at any site. Finally, they reported that calcium intakes had no consistent relationship with BMD.

Our findings must be considered in the context of several limitations. The cohort studied was relatively small, and thus, results could have been influenced by selection bias. The small sample size also limited our ability to control for potential confounding variables. Our subjects were volunteers and 18- to 35-year-old Chinese; thus, the results may not be generalized to other age groups. Although DXA is the gold standard for osteoporosis diagnosis, the 2-dimensional imaging technique does not provide a true volumetric BMD or direct measures of bone strength or the bone compartments (cortical vs. trabecular). Since aBMD is affected by bone size, it may be underestimated in small bones as reported in Asian women [7]. Measures of physical activity and calcium intakes were obtained by self-report questionnaires. Even though these questionnaires have been validated, self-report data tend to overestimate physical activity levels and underestimate caloric intakes. Finally, the cross-sectional design of this study does not allow for a cause and effect relationship between the environmental determinants of bone health. Despite these limitations, this study had several notable strengths, including the addition of male Chinese subjects who are often not included in ethnically related bone studies. This study also used pQCT to evaluate both cortical and trabecular bone parameters at standard sites along the tibia and to examine muscle cross-sectional areas of the thigh.

## 5. Conclusions

Based on the findings of the current study, we determined that relationships between BMD and fat and muscle measures were stronger for women compared to men, that peak bone health is achieved by 25 years of age in Chinese men and women, and differences in bone health were due to differences in body size (height and body weight) rather than sex and accrual phase or age. Although both fat and bone-free lean body mass were related to bone health, Chinese women also showed stronger relationships with muscle function and bone not observed in Chinese men. The implication of these preliminary findings is that Chinese women should focus on improving muscle mass and strength in order to have greater benefits on bone health.

## Figures and Tables

**Table 1:** Subject characteristics (mean ± SD).

Variable	Female (*n* = 25)		Male (*n* = 28)	
	Younger (*n* = 15)	Older (*n* = 10)	Younger (*n* = 15)	Older (*n* = 13)
Age (yr)^*∗∗*^	21.1 ± 1.4^b^	29.7 ± 3.2^a^	21.7 ± 1.7^b^	29.3 ± 2.2^a^
Height (cm)^*∗∗*^	161.6 ± 7.4^a^	164.7 ± 4.3^a^	177.5 ± 4.5^b^	175.4 ± 4.9^b^
Weight (kg)^*∗∗*^	56.8 ± 15.6^a^	58.4 ± 10.9^a^	71.7 ± 6.8^b^	72.0 ± 7.6^b^
BMI (kg/m^2^)	21.5 ± 4.5	21.4 ± 2.7	22.8 ± 2.3	23.4 ± 2.3
SBP (mmHg)^*∗∗*^	104.1 ± 6.9^b^	105.6 ± 10.8^b^	120.4 ± 9.5^a^	122.7 ± 9.7^a^
DBP (mmHg)	72.7 ± 6.4	72.9 ± 10.091	72.4 ± 4.9	78.6 ± 7.6
BPAQ total	22.0 ± 17.2	26.4 ± 39.0	22.0 ± 11.0	22.2 ± 23.5
Total METs	2177.8 ± 1220.8	2151.5 ± 1921.1	2937.8 ± 1638.7	2821.5 ± 3046.3
Calcium (mg/day)	620.4 ± 288.9	520.3 ± 818.3	1012.9 ± 650.3	682.4 ± 307.9
Vitamin D (ng/mL)	18.9 ± 6.6	26.4 ± 39.0	27.2 ± 12.3	24.9 ± 8.2

Yr: years; cm: centimeters; kg: kilograms; m: meters; mmHg: millimeters of mercury; mg: milligrams; mL: milliliters; SD: standard deviation; ^*∗*^*p* ≤ 0.05; ^*∗∗*^*p* ≤ 0.01; a > b.

**Table 2:** Total and regional body composition (mean ± SD).

Variable	Female (*n* = 25)		Male (*n* = 28)	
	Younger (*n* = 15)	Older (*n* = 10)	Younger (*n* = 15)	Older (*n* = 13)
Total body fat (%)^*∗∗*^	34.3 ± 7.2^a^	32.0 ± 4.0^a^	21.9 ± 4.3^b^	20.7 ± 5.3^b^
Total body fat mass (g)^*∗*^	20271 ± 10523^a^	18916 ± 5683^a^	15759 ± 3535^b^	15069 ± 4507^b^
Total body BFLBM (g)^*∗∗*^	33756 ± 5272^b^	37130 ± 5346^b^	53111 ± 5796^a^	54499 ± 6074^a^
Arm fat (%)^*∗∗*^	35.1 ± 6.7^a^	32.6 ± 6.8^a^	17.1 ± 4.7^b^	18.0 ± 5.2^b^
Arm fat mass (g)^*∗*^	2019 ± 951^a^	1839 ± 736^a^	1409 ± 492^b^	1449 ± 440^b^
Arm BFLBM (g)^*∗∗*^	3250 ± 719^b^	3455 ± 720^b^	6369 ± 980^a^	6278 ± 988^a^
Leg fat (%)^*∗∗*^	36.2 ± 6.1^a^	33.4 ± 3.2^a^	21.1 ± 3.3^b^	19.1 ± 4.6^b^
Leg fat mass (g)^*∗∗*^	7462 ± 3535^a^	6841 ± 1847^a^	5209 ± 1121^b^	4662 ± 1344^b^
Leg BFLBM (g)^*∗∗*^	11560 ± 2172^b^	12687 ± 2210^b^	18341 ± 2034^a^	18444 ± 1999^a^
Trunk fat (%)^*∗∗*^	35.0 ± 9.0^a^	33.3 ± 4.9^a^	24.8 ± 5.9^b^	23.6 ± 6.5^b^
Trunk fat mass (g)	9988 ± 5914	9444 ± 3187	8364 ± 2055	8254 ± 2820
Trunk BFLBM (g)^*∗∗*^	16079 ± 2189^b^	17744 ± 2434^b^	24483 ± 3086^a^	25573 ± 3346^a^

%: percent; g: gram; g/cm^2^: gram per square centimeter; BFLBM: bone-free lean body mass; SD: standard deviation; ^*∗*^*p* ≤ 0.05; ^*∗∗*^*p* ≤ 0.01; a > b.

**Table 3:** Regional areal bone density (mean ± SD).

Variable	Female (*n* = 25)		Male (*n* = 28)	
	Younger (*n* = 15)	Older (*n* = 10)	Younger (*n* = 15)	Older (*n* = 13)
L1–L4 aBMD (g/cm^2^)	1.192 ± 0.108	1.170 ± 0.196	1.222 ± 0.084	1.216 ± 0.087
L1–L4 *Z*-score	0.327 ± 0.602	0.140 ± 1.356	0.257 ± 0.736	0.154 ± 0.645
Left femoral neck aBMD (g/cm^2^)^*∗∗*^	0.994 ± 0.190^b^	0.921 ± 0.207^b^	1.121 ± 0.134^a^	1.098 ± 0.189^a^
Left femoral neck *Z*-score	−0.479 ± 0.784	−0.570 ± 1.239	0.214 ± 1.030	0.331 ± 1.331
Right femoral neck aBMD (g/cm^2^)^*∗∗*^	1.011 ± 0.188^b^	0.923 ± 0.229^b^	1.129 ± 0.130^a^	1.080 ± 0.167^a^
Right femoral neck *Z*-score	−0.300 ± 0.813	−0.540 ± 1.409	0.271 ± 0.992	0.185 ± 1.175
Left trochanter aBMD (g/cm^2^)^*∗∗*^	0.762 ± 0.139^b^	0.743 ± 0.212^b^	0.908 ± 0.118^a^	0.865 ± 0.105^a^
Left trochanter *Z*-score	−0.779 ± 0.772	−0.670 ± 1.572	−0.214 ± 1.082	−0.415 ± 0.839
Right trochanter aBMD (g/cm^2^)^*∗∗*^	0.763 ± 0.151^b^	0.752 ± 0.215^b^	0.921 ± 0.121^a^	0.868 ± 0.115^a^
Right trochanter *Z*-score	−0.771 ± 0.882	−0.600 ± 1.619	−0.107 ± 1.089	−0.408 ± 0.948
Left total hip aBMD (g/cm^2^)^*∗∗*^	1.000 ± 0.163^b^	0.948 ± 0.214^b^	1.137 ± 0.131^a^	1.010 ± 0.137^a^
Left total hip *Z*-score	−0.086 ± 0.865	−0.240 ± 1.456	0.271 ± 0.875	0.162 ± 0.844
Right total hip aBMD (g/cm^2^)^*∗∗*^	1.010 ± 0.163^b^	0.949 ± 0.217^b^	1.140 ± 0.124^a^	1.087 ± 0.144^a^
Right total hip *Z*-score	0.000 ± 0.855	−0.240 ± 1.480	0.314 ± 0.832	0.077 ± 0.916

L1–L4: 1^st^ to 4^th^ lumbar vertebrae; aBMD: areal bone mineral density; g/cm^2^: gram per square centimeter; SD: standard deviation; ^*∗*^*p* ≤ 0.05; ^*∗∗*^*p* ≤ 0.01; a > b.

**Table 4:** pQCT bone quantity and quality (mean ± SD).

Variable	Female (*n* = 25)		Male (*n* = 28)	
	Younger (*n* = 15)	Older (*n* = 10)	Younger (*n* = 15)	Older (*n* = 13)
*TIBIA 4% site*				
Total vBMD (mg/cm^3^)^*∗∗*^	299 ± 37^b^	286 ± 57^b^	341 ± 31^a^	343 ± 41^a^
Trabecular vBMD (mg/cm^3^)^*∗∗*^	252 ± 37^b^	233 ± 59^b^	297 ± 28^a^	288 ± 31^a^
Total BSI (mg^2^/mm^4^)^*∗∗*^	89 ± 29^b^	88 ± 36^b^	144 ± 26^a^	143 ± 26^a^
Trabecular BSI (mg^2^/mm^4^)^*∗∗*^	52.5 ± 21.5^b^	48.1 ± 32.9^b^	89.7 ± 17.6^a^	81.6 ± 13.6^a^

*TIBIA 38% site*				
Total vBMD (mg/cm^3^)	880 ± 61	898 ± 52	905 ± 61	903 ± 81
Cortical vBMD (mg/cm^3^)	1184 ± 19	1201 ± 17	1189 ± 19	1190 ± 23
Cortical SSI (mm^3^)^*∗*^	1583 ± 369^b^	1534 ± 396^b^	1669 ± 510^a^	2012 ± 641^a^

*TIBIA 66% site*				
Total vBMD (mg/cm^3^)	671 ± 66	671 ± 58	698 ± 67	635 ± 75
Cortical vBMD (mg/cm^3^)^*∗*^	1142 ± 20^b^	1169 ± 12^a^	1151 ± 21^b^	1148 ± 13^a^
Cortical SSI (mm^3^)	2451 ± 642	2328 ± 549	2543 ± 773	2670 ± 655

vBMD: volumetric bone mineral density; pQCT: peripheral quantitative computed tomography; BSI: bone strength index; SSI: strength-strain index; SD: standard deviation; ^*∗*^*p* ≤ 0.05; ^*∗∗*^*p* ≤ 0.01; a > b; older > younger.

**Table 5:** Muscle strength, power, and cross-sectional area (mean ± SD).

Variable	Female (*n* = 25)		Male (*n* = 28)	
	Younger (*n* = 15)	Older (*n* = 10)	Younger (*n* = 15)	Older (*n* = 13)
1RM leg press (kg)^*∗∗*^	104.3 ± 31.6^b^	96.6 ± 26.1^b^	165.1 ± 18.6^a^	230.0 ± 116.1^a^#
R handgrip strength (kg)^*∗∗*^	25.3 ± 4.7^b^	27.4 ± 2.9^b^#	41.3 ± 4.3^a^	44.7 ± 5.8^a^#
L handgrip strength (kg)^*∗∗*^	24.1 ± 3.9^b^	25.8 ± 3.5^b^	38.9 ± 4.9^a^	41.5 ± 6.0^a^
Maximal handgrip (kg)^*∗∗*^	25.6 ± 1.1^b^	27.7 ± 0.9^b#^	41.9 ± 4.6^a^	45.0 ± 5.7^a#^
Average jump time (s)^*∗∗*^	0.50 ± 0.04^b^	0.52 ± 0.03^b^	0.62 ± 0.04^a^	0.63 ± 0.05^a^
Average jump height (cm)^*∗∗*^	31.8 ± 5.1^b^	33.3 ± 3.3^b^	46.9 ± 6.0^a^	49.5 ± 7.1^a^
Maximal jump power (W)^*∗∗*^	656.1 ± 189.9^b^	761.0 ± 184.1^b^	972.1 ± 106.9^a^	974.1 ± 184.4^a^
Muscle cross-sectional area (mm^2^)^*∗∗*^	5941 ± 1136^b^	6980 ± 980^b^#	7980 ± 96^a^	8812 ± 974^a^#

1RM: repetition maximum; kg: kilogram; R: right; L: left; s: seconds; cm: centimeters; W: watts; mm^2^: square millimeters; SD: standard deviation; ^*∗∗*^*p* ≤ 0.01; a > b; # older > younger.

**Table 6:** Pearson correlation coefficients for age, height, body composition, muscle performance, and bone characteristics in males.

Males (*n* = 28)	L1–L4 aBMD	RFN aBMD	RTroc aBMD	RHip aBMD	4% trab vBMD	4% tot BSI	4% trab BSI	38% cort vBMD	38% cort SSI	66% cort vBMD
Age	0.14	−0.06	−0.13	−0.06	0.08	0.08	−0.07	0.14	0.12	−0.03
Height	0.53^*∗∗*^	0.45^*∗*^	0.28	0.35	0.30	0.19	0.25	−0.03	−0.04	0.13
Weight	0.27	0.50^*∗∗*^	0.34	0.49^*∗∗*^	0.08	0.41^*∗*^	0.13	−0.06	0.05	−0.01
BMI	0.01	0.28	0.20	0.32	−0.05	0.33	0.23	−0.04	0.06	−0.09
% fat	0.05	0.07	0.07	0.08	0.11	0.09	0.10	−0.01	−0.33	−0.12
Fat mass	0.16	0.26	0.19	0.27	0.14	0.27	0.15	−0.03	−0.26	−0.10
Total BFLBM	0.20	0.38^*∗*^	0.25	0.37	−0.03	0.28	0.02	−0.04	0.25	0.07
1RM leg press	0.31	0.31	0.27	0.32	−0.11	0.04	−0.02	0.01	−0.15	−0.13
Max HG	0.31	0.24	0.16	0.22	−0.05	0.13	−0.08	0.02	0.00	−0.15
Max power	0.30	0.59^*∗∗*^	0.46^*∗∗*^	0.59^*∗∗*^	0.17	0.38^*∗*^	0.08	0.09	−0.07	0.13

^*∗*^ *p* ≤ 0.05; ^*∗∗*^*p* ≤ 0.01. BMI: body mass index; BFLBM: bone-free lean body mass; 1RM: 1-repetition maximum; Max HG: maximal handgrip; Max Power: maximal jump power; L1–L4: 1^st^ to 4^th^ lumbar vertebrae; aBMD: real bone mineral density; RFN: right femoral neck; RTroc: right trochanter; RHip: right total hip; trab: trabecular; cort: cortical; vBMD: volumetric bone mineral density; BSI: bone strength index; SSI: strength-strain index.

**Table 7:** Pearson correlation coefficients for age, height, body composition, muscle performance, and bone characteristics in females.

Females (*n* = 25)	L1–L4 aBMD	RFN aBMD	RTroc aBMD	RHip aBMD	4% trab vBMD	4% tot BSI	4% trab BSI	38% cort vBMD	38% cort SSI	66% cort vBMD
Age	−0.20	−0.40	−0.23	−0.33	−0.43^*∗*^	−0.26	−0.29	−0.36	−0.11	0.60^*∗∗*^
Height	0.51^*∗∗*^	0.51^*∗∗*^	0.59^*∗∗*^	0.53^*∗∗*^	0.41^*∗*^	0.61^*∗∗*^	0.64^*∗∗*^	−0.01	0.26	−0.03
Weight	0.62^*∗∗*^	0.69^*∗∗*^	0.73^*∗∗*^	0.72^*∗∗*^	0.66^*∗∗*^	0.79^*∗∗*^	0.80^*∗∗*^	−0.25	0.46^*∗*^	−0.32
BMI	0.57^*∗∗*^	0.65^*∗∗*^	0.66^*∗∗*^	0.67^*∗∗*^	0.65^*∗∗*^	0.74^*∗∗*^	0.74^*∗∗*^	−0.29	0.46^*∗*^	−0.38
% fat	0.36	0.48^*∗*^	0.44^*∗*^	0.49^*∗*^	0.45^*∗*^	0.52^*∗∗*^	0.52^*∗∗*^	−0.32	0.45^*∗*^	−0.44^*∗*^
Fat mass	0.53^*∗∗*^	0.62^*∗∗*^	0.62^*∗∗*^	0.65^*∗∗*^	0.60^*∗∗*^	0.69^*∗∗*^	0.69^*∗∗*^	−0.28	0.47^*∗*^	−0.39
Total BFLBM	0.60^*∗∗*^	0.57^*∗∗*^	0.69^*∗∗*^	0.61^*∗∗*^	0.59^*∗∗*^	0.77^*∗∗*^	0.78^*∗∗*^	−0.09	0.39	−0.09
1RM leg press	0.43^*∗*^	0.55^*∗∗*^	0.57^*∗∗*^	0.56^*∗∗*^	0.57^*∗∗*^	0.49^*∗*^	0.50^*∗*^	0.04	0.09	−0.17
Max HG	0.12	0.11	0.25	0.18	0.25	0.48^*∗*^	0.38	−0.16	0.29	−0.20
Max power	0.70^*∗∗*^	0.64^*∗∗*^	0.75^*∗∗*^	0.70^*∗∗*^	0.70^*∗∗*^	0.82^*∗∗*^	0.82^*∗∗*^	−0.08	0.47^*∗*^	−0.12

^*∗*^ *p* ≤ 0.05; ^*∗∗*^*p* ≤  0.01. BMI: body mass index; BFLBM: bone-free lean body mass; 1RM: 1 repetition maximum; Max HG: maximal handgrip; Max Power: maximal jump power; L1–L4: 1^st^ to 4^th^ lumbar vertebrae; aBMD: areal bone mineral density; RFN: right femoral neck; RTroc: right trochanter; RHip: right total hip; trab: trabecular; cort: cortical; vBMD: volumetric bone mineral density; BSI: bone strength index; SSI: strength-strain index.

## Data Availability

The data used to support the findings of this study are restricted by the University of Oklahoma Health Sciences Center IRB in order to protect participant privacy. Data may be available in aggregate form from the corresponding author upon request.
